# Comments from the Editors on the Special Issue “Assessment and Treatment of Addictions: New Tools for Old Problems”

**DOI:** 10.3390/jcm8101717

**Published:** 2019-10-17

**Authors:** Pablo Barrio, Laia Miquel, Antoni Gual

**Affiliations:** 1 Grup de Recerca en Addiccions Clínic (GRAC), Addiction Unit Hospital Clínic of Barcelona, Department of Psychiatry, 08036 Barcelona, Spain; PBARRIO@clinic.cat (P.B.); laiamiqueldemontagut@gmail.com (L.M.); 2 Institut d’Investigacions Biomèdiques August Pi i Sunyer (IDIBAPS), 08036 Barcelona, Spain

**Keywords:** addiction, craving, treatment, assessment instruments, digital health

## Abstract

New conceptual and technological solutions have been proposed to solve addictive disorders and will be presented in the future. In this Special Issue, we present some of the new assessment tools and treatment options for internet addiction, alcohol, cannabis, cocaine, and gambling disorders.

Addiction represents an enormous challenge to society. Worldwide, it has been estimated that alcohol, tobacco, and illicit drugs were responsible for more than 10 million deaths [1], with a higher impact in developed countries where substance use disorders have been identified as responsible for life expectancy reversals [2]. Societal and medical responses to the problem are far from optimal, but the appearance of new technologies offers room for improvement, with lots of new initiatives launched and developed. This special issue is intended to describe and discuss how these new tools are helping to improve the assessment and treatment of such old problems (addictive disorders), covering a wide diversity of novelties that are being applied in the field.

Digital health entails the possibility to overcome existent problems around addictive disorders like stigmatization, addiction identification, treatment access, adherence and treatment efficacy by facilitating the improvement in knowledge, assessment, diagnosis, and treatment of addictive disorders. Assessment is one of the areas where new solutions have probably reached furthest. Think, for example, about transdermal sensors and ecological momentary assessment: a clear example of how new technologies can reach the core of a patient’s drinking pattern. In this special issue, Barrio et al. investigate patients’ attitudes towards transdermal sensors in real clinical settings. Digital technologies might be useful to assess brain damage, and they bring us closer to understanding the mechanism(s) underlying addiction. Herreros et al. present a visuomotor rotation task which might be the first step towards developing a useful tool for the detection of cerebellum dysfunction by assessing alterations in implicit learning among chronic cannabis users. New technologies have opened up the possibility of not only assessing patients “right here right now” but also of creating new realities, or should we better say virtual realities? Ghiţă et al. show us that virtual reality can enhance the assessment of alcohol-induced craving and anxiety. Technological advances in neuroimaging and genetics have allowed deepening in the understanding of some learning processes involved in substance use disorders. Garbusow et al. provide knowledge on how important processes, such as instrumental responses to relevant stimuli, are influenced by drinking patterns. In this same line, Heinz et al. conduct a review that starts with how Pavlovian and instrumental learning mechanisms interact in drug addiction and finishes with how these learning mechanisms and their respective neurobiological correlates can contribute to losing versus regaining control over drug intake.

Paradoxically enough, new technologies do also have their own risks. Take, for example, internet gaming disorder. Ryu et al. present the development of a new assessment instrument for internet addiction, the Diagnostic Interview for Internet Addiction. And keeping in mind that new psychometric instruments are also new solutions to old problems, Chen et al. investigate an interesting phenomenon in addiction: the intensity of memory addiction. They present us the development of the Addiction Memory Intensity Scale.

In the treatment area, this special issue offers an interesting combination of modalities: newly designed pharmaceutical compounds (nalmefene), naturally occurring psychoactive substances (cannabidiol), and non-pharmacological, biological therapies (rTMS). Barrio et al. report the main effectiveness analysis of a phase-IV study conducted among alcohol dependent outpatients taking nalmefene, the only approved medication for alcohol reduction aims. The use of rTMS is presented by Cardullo et al. in a sample of cocaine and gambling patients. The stimulation of the left dorsolateral prefrontal cortex yields promising results. Finally, Batalla et al. review the potential use of cannabidiol in addictive and comorbid psychotic disorders, pointing to a prominent role in the treatment of cannabis addiction.

Scientific advances and new technologies are providing new tools that let us expand our knowledge, and improve diagnosis and treatment of addictive behaviors, presenting us with opportunity for success and giving people back their health.
